# The Evolution of Structural Defects under Irradiation in W by Molecular Dynamics Simulation

**DOI:** 10.3390/ma16124414

**Published:** 2023-06-15

**Authors:** Ruxin Zheng, Wujing Xuan, Junjun Xie, Shasha Chen, Liuqing Yang, Liang Zhang

**Affiliations:** 1 International Joint Laboratory for Light Alloys (MOE), College of Materials Science and Engineering, Chongqing University, Chongqing 400044, China; 2 Shenyang National Laboratory for Materials Science, Chongqing University, Chongqing 400044, China

**Keywords:** irradiation, collision cascade, structural defects, grain boundary, molecular dynamics

## Abstract

Tungsten (W) can be used in plasma-facing components in a fusion reactor because of its excellent radiation resistance. Some studies have found that nanocrystalline metals with a high density of grain boundary show a higher ability to resist radiation damage compared to conventional coarse-grained materials. However, the interaction mechanism between grain boundary and defect is still unclear. In the present study, molecular dynamics simulations were carried out to explore the difference of defect evolution in single-crystal and bicrystal W, while the effects of temperature and the energy of the primary knocked atom (PKA) were taken into account. The irradiation process was simulated at the temperature range of 300 to 1500 K, and the PKA energy varied from 1 to 15 keV. The results show that the generation of defects is more sensitive to the energy of PKA than temperature; the number of defects increases at the thermal spike stage with the increase of the PKA energy, but the correlation with temperature is not strong. The presence of the grain boundary prevented the recombination of interstitial atoms and vacancies during the collision cascades, and the vacancies were more likely to form large clusters than interstitial atoms in the bicrystal models. This can be ascribed to the strong segregation tendency of the interstitial atoms to grain boundaries. The simulations provide useful information for understanding the role of grain boundaries in the evolution of irradiated structural defects.

## 1. Introduction

The irradiation of metals under high-energy particles produces interstitial atoms and vacancies that can form many defect structures such as voids, stacking fault tetrahedron, and dislocation loops [1,2,3,4]. The evolution process of defects may cause a series of microstructure changes such as swelling, hardening, or amorphization, which may lead to the degradation of material properties which then severely affect the reliability and lifetime of the material for nuclear energy applications [5,6,7,8]. With the rise of nanomaterials, researchers have found that grain boundaries (GBs) can serve as effective sinks for absorbing and annihilating radiation-induced point defects [9,10,11,12], and this can effectively reduce the radiation damage to materials. Nanostructured metals have demonstrated enhanced radiation tolerance because of their high-density GBs compared to their bulk counterparts [13]. In conventional metals, the interstitial atoms quickly move to the surface and cause swelling, but the vacancies move slowly and are more likely to form immobile voids in the bulk region that impede dislocation motion and cause hardening and embrittlement of the material [14,15]. When there are more GBs in the crystal such as nanocrystalline metals, the interstitial atoms can be captured by GBs easily, and then be emitted to recombine with the nearby vacancies, which has shown high ability of resistance to irradiation. In addition, the interaction between GBs and defects can be observed visually by computer simulations [16]. Molecular dynamics (MD) methods were used to simulate at nanometer and picosecond levels, which is suited to the research of the radiation damage of nuclear materials [17,18].

Tungsten (W) based alloys are seen as the prime candidates for use in plasma-facing components in fusion reactors because of their high melting temperature, high thermal conductivity, and low sputtering erosion [19,20,21,22]. Although tungsten-based alloys have many excellent properties, there are still some problems in the practical application of thermonuclear fusion devices, such as low temperature brittleness, radiation sensitivity, and high temperature oxidation [23]. Many researchers have carried out series of works on the radiation effects on W to explain the mechanism and improve these problems [24,25,26]. In terms of experimentation, Jäger and Wilkens [24] investigated the formation of dislocation loops in W and explained it by the elastic interaction of the growing loops with the adjacent specimen surface. Šestan et al. [26] investigated the effect of helium implantation on microstructure evolution of polycrystalline W/W_2_C composite consolidated. For computational simulations, the method of MD has been broadly applied to study the collision cascades [27,28,29]. Domínguez-Gutiérrez investigated the formation damage in W and found the production of the Frenkel pair increases as a function of the temperature due to thermally activated mechanisms [27]. In the study by Sand et al. [29], collision cascades in W were observed, and they found the fractal nature of the cascades gave rise to a scale-less power-law-type size distribution of defect clusters.

Most of the previous MD simulation works of W irradiation pay more attention to the process of irradiation-induced defects and the influence of external conditions (such as temperature, PKA energy, etc.) on the irradiation results. However, grain boundaries, as an important microstructure feature of materials, are rarely considered for their effect on irradiation. The absorption effect of GBs on defects has been well demonstrated [16], but the microscopic mechanism of the interaction between GBs and point defects has been less studied. In this research, the evolution of structural defects of W under irradiation with different temperatures and PKA energies were studied, and more importantly, the effect of various GBs on the formation of structural defects investigated. To understand the effect of GBs, the simulations of four bicrystal models with different GBs and their corresponding single-crystals were studied. We elucidated the mechanisms of defect generation and evolution, including point defects and defect clusters near GBs and compared the results to those of single-crystals.

## 2. Methods

### 2.1. Model and Simulation

**Figure 1:**
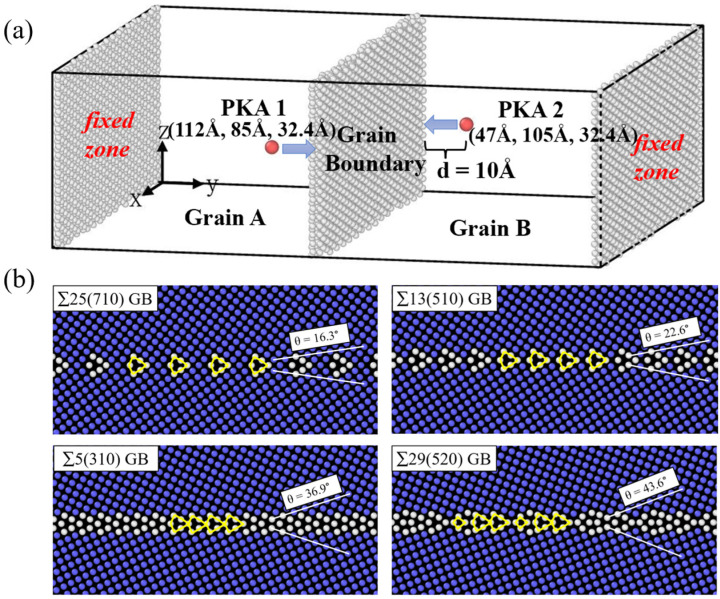
(**a**) Schematic of the simulation model. Atoms with perfect BCC structure are removed; the red atoms are the PKA atoms. (**b**) The atomic structure of the four GBs studied in this work. Atoms are colored according to the common neighbor analysis (CNA) parameter, the blue atoms are in perfect BCC structure, and the white atoms are in the GB plane. The misorientation angles of the GB are indicated, and the GB structural units are outlined by the yellow lines.

The MD simulations were performed with the Large-scale Atomic/Molecular Massively Parallel Simulator (LAMMPS) [30]. We created a cuboid simulation box with about 128,000 atoms, which only contained the element W in a well-defined body-centered cubic (BCC) crystal. Depending on the GB structure, the total number of atoms in each model has a certain difference. However, the overall structure is consistent, which did not affect our subsequent simulation results. Taking the bicrystal containing Σ5(310) GB as an example, the GB was built by creating two separate crystals with the desired crystallographic orientations and joined along a plane normal to the y-direction. Similarly, the other GBs were constructed in the same way. The grains were sandwiched between two slabs in which the atoms were fixed in their perfect lattice positions relative to one another while the atoms in the fixed zone did not participate in the MD simulations [31]. In all simulations of GB structures, the PKA velocities were along the y-direction. PKA 1 with a kinetic energy of 15 keV was introduced to generate the radiation damage, while PKA 2 with the same energy came from the opposite direction. The bicrystal model and the initial positions of the two PKAs relative to the coordinate origin are indicated in Figure 1a.

Four symmetric tilt GBs were investigated in this study, namely Σ25(710) (θ = 16.3°) GB, Σ13(510) (θ = 22.6°) GB, Σ5(310) (θ = 36.9°) GB, and Σ29(520) (θ = 43.6°) GB. The detailed GB structures are shown in Figure 1b where all the GBs contain topologically identical structural units, as outlined by the yellow line. They differ only by the distance separating the structural units and by their positions relative to the GB plane. Here, we tentatively defined the Σ25 and Σ13 GBs as the low-angle GBs, because the structure of the two GBs can be described as an array of discrete perfect dislocations, while the dislocation cores are formed by the structural unit. Accordingly, the Σ5 and Σ29 GBs were regarded as the high-angle GBs in this study. For comparison, four single-crystal models with orientations of (710), (510), (310), and (520) were also constructed. The size of the simulated box of the single-crystal models is the same as their corresponding bicrystal models. The detailed information on the simulation models is listed in Table 1. There are subtle differences in model sizes for GB structures, which leads to a difference in the number of atoms.

In order to investigate the temperature effect on the production of structural defects, simulations were performed at different temperatures (T = 300, 500, 800, 1000, 1300, and 1500 K). In addition, for exploring the effects of the incident energy of PKA on the defect production, different PKA energies were tested, ranging from 1 to 15 keV with an interval of 2 keV. The simulation model first performed energy minimization and then relaxed within the NPT ensemble (constant number of atoms, pressure, and temperature) at the corresponding temperature for 10 ps to obtain the equilibrium structure. Then, the NVE ensemble (constant number of atoms, volume, and total energy) was employed to simulate the dynamic process of the incident PKA. The potential selected in this work is the embedded-atom method potential developed by Marinica [32], which was successfully applied in many previous works to investigate the defects under the irradiation of W [33,34,35,36,37]. The Open Visualization Tool (OVITO) was used for defect analysis and visualization of the simulation results [38].

### 2.2. Structure Defect Analysis

The Wigner–Seitz cells method was used to identify vacancies and interstitials, and also could be used to count the number of defects [39]. The method requires two configurations of the atomistic system as input: the reference configuration and the displaced configuration. The reference configuration was a perfect crystal lattice where every site was occupied by exactly one atom. The displaced configuration was an arrangement of real atoms that needed to be analyzed. The atoms from the reference configuration would be denoted as sites, while atoms from the displaced configuration would simply be referred to as atoms. The sites occupied by no atoms are defined as vacancies, while the sites occupied by more than one atom are called interstitials, and the atoms occupying them are defined as interstitial atoms. The number of vacancies and interstitial atoms are counted by an algorithm. In addition, we also analyzed the sizes and distribution of the defect clusters involved in this work. Cluster analysis was done by calculating the distance between each defect and all other defects. A cluster is defined as a set of connected particles, each of which is within the (indirect) reach of the other particles in the same cluster. Therefore, any two particles form the same cluster which fulfills the selected neighboring criterion, and if not, the two particles belong to different clusters. In previous studies, all defects that were within a fixed cut-off radius from each other were interpreted to be a part of the same defect cluster, and the distance between the atom and its second-nearest-neighbor was usually selected as the cut-off radius [40,41]. For BCC tungsten, the distance that is set as the cut-off radius, is just equal to its lattice constant (3.16 Å). Two or more vacancies and interstitial agglomeration were treated as clusters.

## 3. Results and Discussion

### 3.1. The Effect of Temperature

**Figure 2:**
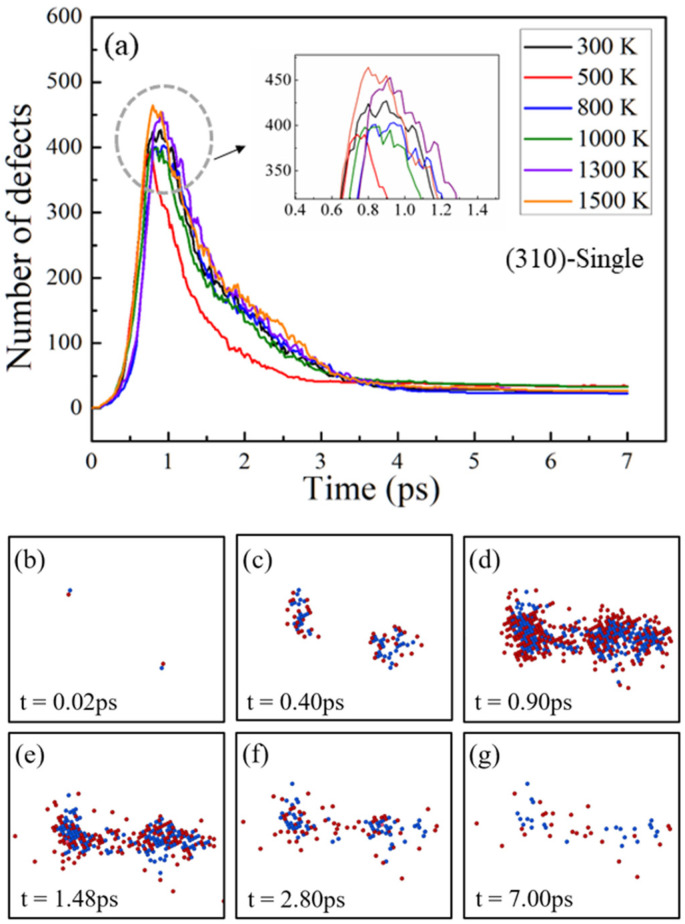
(**a**) The number of point defects produced as a function of simulation time in (310) single-crystal at different temperatures with PKA energy of 9 keV. (**b**–**g**) Snapshots of defect distributions in the (310) single-crystal at 300 K. The red atoms are interstitial atoms, and the blue atoms are vacancies.

Figure 2a shows the number of defects produced by the irradiation in single-crystals at different temperatures. The primary energy of PKA 1 and PKA 2 was set as 9 keV. According to the simulation results, a number of interstitial atoms and vacancies were produced by the dynamic motion of the PKAs. The number of defects increased rapidly in a short time. Then it reached a maximum value, which is the peak stage of the collision cascade, also known as the thermal spike stage [10,12]. As the simulation time progressed, the interstitial atoms recombined with the vacancies, leaving a few structural defects, and the number of defects no longer changed significantly, which is the stable stage of the collision cascade. It takes about 0.9 ps for the number of defects to reach the peak stage at 300 K, while it takes about 0.7 ps at 500 K. However, with the increase of temperature, the time required for the number of defects to reach the maximum value did not observe an obvious rule. At different temperatures, the evolution of defects in the collision cascade process tends to be consistent; that is, a large number of defects are generated first and then gradually reduced. Similar results were found in Nb and α-Fe; the number of defects in the crystal increased to the maximum value and then decreased to a stable value [42,43]. The temperature change caused the difference in the number of defects, but it did not play a significant role in the overall evolution of the irradiated structural defects.

Figure 2b–g shows the distribution of the defects in the (310) single-crystal at different simulation times at 300 K. As the PKAs transfer energy to the surrounding atoms during irradiation, the number of defects produced in the crystal increases gradually. The number of defects produced within the crystal reaches a maximum at 0.9 ps. In addition, vacancies generated by irradiation are distributed in the central region of the collision cascade, forming an atom-poor region. The interstitial atoms are distributed at the edge of the cluster of vacancy atoms, forming a shell of interstitial atoms, as shown in Figure 2d, where the blue atoms are wrapped in the surrounding red atoms. In Figure 2e–g, the number of defects decreases as interstitial atoms recombine with vacancies and reaches a steady value at 7 ps. The simulation results show that the evolution of structural defects in single-crystals under irradiation includes three stages, namely, rapid increase stage, thermal spike stage, and stable stage.

**Figure 3:**
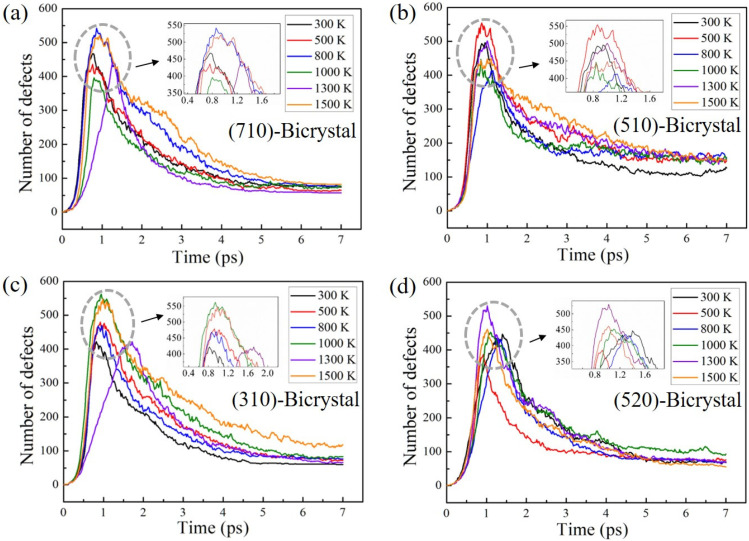
The number of point defects produced as a function of simulation time at different temperatures of bicrystal models with (**a**) Σ25(710) GB, (**b**) Σ13(510) GB, (**c**) Σ5(310) GB, and (**d**) Σ29(520) GB. The PKA energy is set as 9 keV.

To understand the effects of GBs on the defects, the number of defects in the bicrystal models with four GB structures were investigated, and the results are shown in Figure 3. The variation of defects in bicrystals has shown a similar trend as that in single-crystals. The number of defects produced near GBs reaches a maximum value and finally drops down to a stable value. However, for different GB structures, the maximum value of the defects varies as the temperature rises. As shown in Figure 3a,b, for the low-angle GBs, the maximum value of defects is largest for Σ25(710) GB at the lower temperature of 800 K and for Σ13(510) GB at 500 K. In contrast, Figure 3c,d shows that the maximum value of defects near the high-angle GBs is largest at the higher temperature of 1000 K for Σ5(310) GB and 1300 K for Σ29(520) GB, respectively. In addition, there are differences in the number of residual defects in the stable stage near different GBs. On the one hand, the mobility of the atoms increases with increasing temperature, and the interstitial atoms recombine with the vacancies more easily. On the other hand, the four GB structures differ in their absorption of interstitial atoms and vacancies, resulting in differences in the number of defects remaining within the crystal after the irradiation cascade.

**Figure 4:**
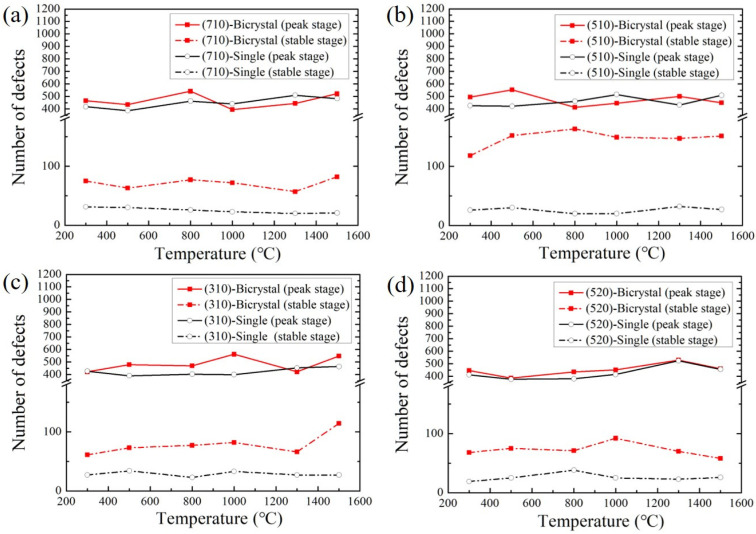
The number of defects at the peak stage and stable stage in single-crystal models and bi crystal models with crystallographic orientations of (**a**) (710), (**b**) (510), (**c**) (310), and (**d**) (520) at different temperatures.

**Figure 5:**
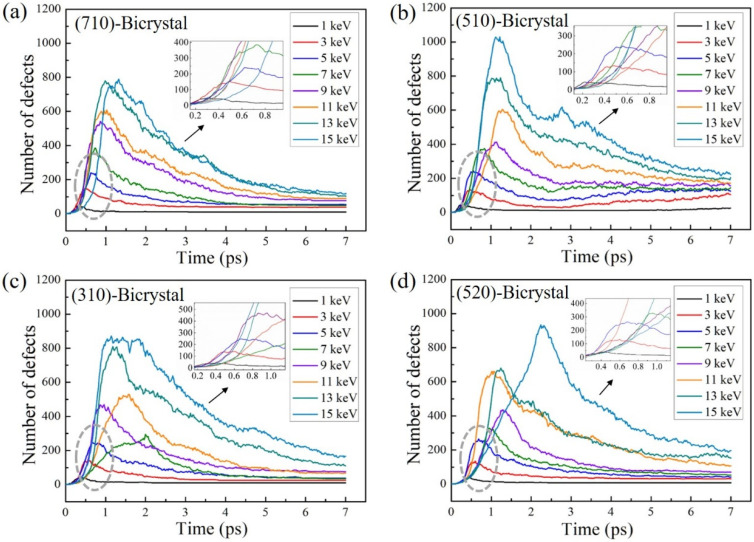
The number of point defects produced as a function of simulation time with different PKA energies of bicrystal models with (**a**) Σ25(710) GB, (**b**) Σ13(510) GB, (**c**) Σ5(310) GB, and (**d**) Σ29(520) GB. The temperature is set as 800 K.

The maximum and stable value of defects produced near GBs at different temperatures are counted in Figure 4. According to the results, the maximum and stable values of defects are not linearly increased by increasing temperature, whether it is a single crystal or a bicrystal. In previous studies, the researchers found that the stable value of defects is reduced with increasing temperature in a single crystal of tungsten [44,45]. However, in their studies, only a limited temperature range two (363 K and 600 K) or three (100 K, 500 K and 900 K) temperatures were investigated. Therefore, the influence of temperature on simulation results needs to be further verified over a wider temperature range. For example, Sahi et al. [44] found that the defects did not increase with increasing temperature in the study of α-iron under irradiation. In addition, there are few studies on the variation law of the number of defects in bicrystals with increasing temperature. In our study, with increasing temperature, the number of defects produced in the crystals at the peak stage was between 400 to 500, while the number of surviving defects in different crystals at the end of collision cascades varied greatly from 0 to 200. For example, the number of defects at the peak stage in Σ25(710) GB was 467 and at the stable stage 75 at 300 K (Figure 5a). For the (710) single-crystal, the number of defects produced at the peak stage and which survived at the stable stage was largest at 800 K of all the temperatures, except at 1500 K, where more defects survived. By comparison, the maximum value of defects in the Σ25(710) GB was greater than that in the (710) single-crystal at other temperatures except for 1000 and 1300 K, and more defects survived in the bicrystal than the single-crystal at all temperatures. Similarly, the number of residual defects near the other three GBs was higher than that of the single-crystal, as shown in Figure 5b–d. Among the four GB structures, the stable value of defects near the Σ13(510) GB was much higher than that of the other three GBs, which is more likely to produce structural defects (such as clusters and voids) and show a lower ability to the resistance of irradiation damage. Therefore, due to the strong segregation effect of interstitial atoms at the GBs, the recombination of interstitial atoms and vacancies was prevented, resulting in a higher number of defects in the bicrystals than in the single-crystals at the initial stage of irradiation.

### 3.2. The Effect of PKA Energy

**Figure 6:**
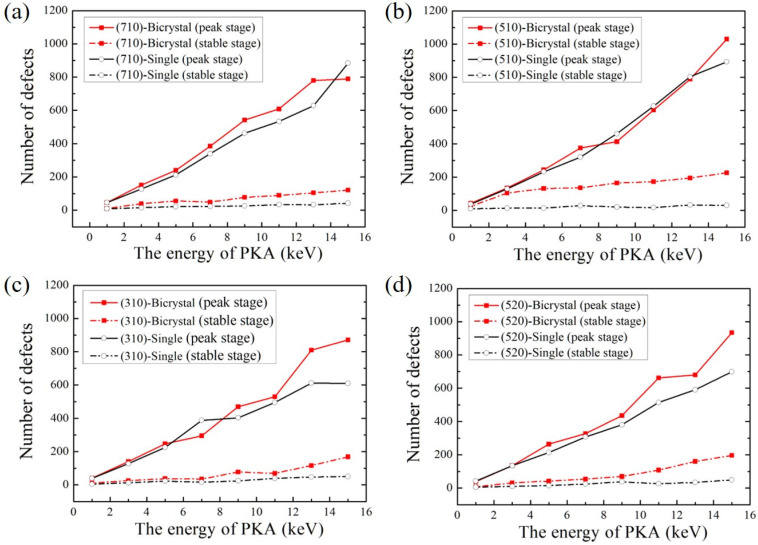
The number of defects at the peak stage and stable stage in single-crystal models and bicrystal models with crystallographic orientations of (**a**) (710), (**b**) (510), (**c**) (310), and (**d**) (520) at different PKA energies.

The variation of defects near the four GBs with different PKA energies (E_PKA_) are plotted in Figure 5. The defect variation is similar to the above simulations which goes through a rapid increase stage, a thermal spike stage, and a stable stage. When the PKA incident is at lower energy, the collision cascade occurs only in a small area and ends quickly. For example, when E_PKA_ = 1 keV, the number of defects quickly reaches the maximum value at 0.3 ps and only a few defects remain near the Σ25(710) GB at the end of simulation for 7 ps. When E_PKA_ = 15 keV, it needs about 1.5 ps to reach the maximum value. The generation and annihilation of defects take more time with the increase of PKA energy. Similar results are observed in the simulation cases of the other GBs shown in Figure 6b–d. It is worth noting that the maximum value of the defects in Σ13(510) GB is overall higher than the other three GBs, indicating that the GB structure can affect the generation of defects and cause a difference in the number of defects near the GBs. With the increase of PKA energy, more defects are produced, and some interstitial atoms migrate to distant positions and are difficult to recombine with vacancies, resulting in more defects remaining in the crystal. The simulation results show that with the increase of PKA energy, the number of defects increases during the whole collision cascade process, and more defects are generated inside the crystal.

**Figure 7:**
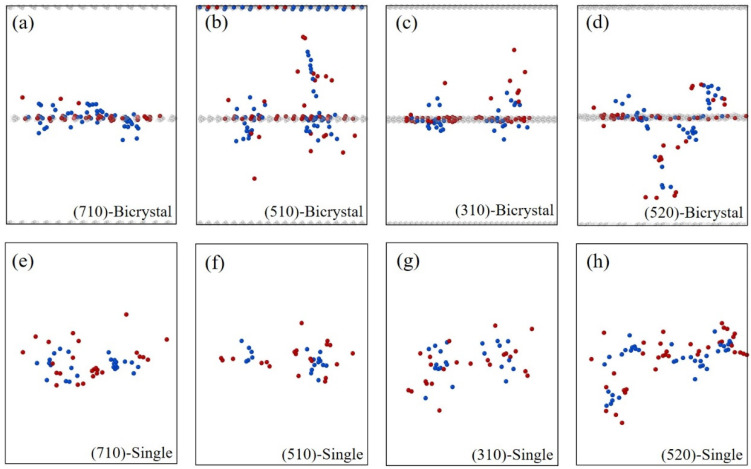
Snapshots of defect distributions after 7 ps simulation of the collision cascades in bicrystals with different GBs (**a**–**d**) and single-crystals with different orientations (**e**–**h**). The red atoms are interstitial atoms, the blue atoms are vacancies, and the gray atoms are GBs. All simulations are conducted with the case of E_PKA_ = 9 keV at a temperature of 800 K.

The number of defects at the peak stage and stable stage in single-crystals and bicrystals are counted in Figure 6. It was found that the number of defects was positively correlated with PKA energy, and more defects were generated and retained in both single and bicrystal models with the increase of PKA energy. A similar conclusion was also obtained in the study of Park et al. who found more defects remain at higher energies [46]. As shown in Figure 6a, there are more defects in the bicrystal with Σ25(710) GB in both peak and stable stages compared with the (710) single-crystal. When the E_PKA_ = 15 keV, the maximum value of defects in the bicrystal is lower than that in the single-crystal. Similar scenarios are observed for Σ13(510) GB at E_PKA_ = 9, 11, and 13 keV, Σ5(310) GB at E_PKA_ = 7 keV, and Σ29(520) GB at E_PKA_ = 1 keV, as shown in Figure 7b–d. It is worth noting that the number of defects produced near the low-angle GBs is less than that of the corresponding single-crystals at higher energies at the E_PKA_ ≥ 9 keV. On the contrary, for the high-angle GBs, there are more defects produced at lower energies at the E_PKA_ ≤ 7 keV in single-crystals. In most cases as shown in Figure 6, more defects were retained in the bicrystal models than in single-crystals, indicating that the existence of GB prevents the recombination of interstitial atoms with vacancies at the initial stage of the collision cascade. By comparing the results of Figure 4 and Figure 6, while the number of defects fluctuates with the change of temperature, a linear relationship with the PKA energy can be shown; that is, the energy of PKA plays a significant role in the evolution of the irradiated crystal defects.

### 3.3. The Distribution of Clusters near GBs

Figure 7 shows the distributions of defects after 7 ps simulation of the collision cascades in bicrystal and single-crystal models. As shown in Figure 7a–d, most of the interstitial atoms are biased at GBs, while most of the vacancies are distributed in the bulk region. For the single-crystals shown in Figure 7e–h, interstitial atoms and vacancies are randomly distributed in the bulk region. The simulations indicate that the GBs can serve as a defect sink for absorbing interstitial atoms as compared with the single-crystal. The results are similar to previous work [14,37], where it was reported that the bulk region is vacancy-rich compared to the interstitial-rich GB region. The bias-absorption for interstitials by GB is due to the difference between the migration energy barriers of interstitials and vacancies in the bulk region [14,47,48]. The vacancies can be considered stationary compared with the higher mobility of interstitial atoms at the time scale of MD simulation [12]. Therefore, the interstitial atoms are not easy to recombine with vacancies because of their strong segregation tendency to GBs. In addition, different from the stress-driven migration of GBs [18,49], the GB models in this study are stationary without external stress; it is difficult to re-release interstitial atoms after their absorption by GBs, which causes more residual defects in bicrystal models than in the single-crystals. In Figure 7a, the residual structural defects in bicrystal containing Σ25(710) GB are distributed close to the GB. For Σ13(510) GB, some of the interstitial atoms and vacancies migrate to positions far from the GB, as shown in Figure 7b. Similarly, there are also some interstitial atoms and vacancies that are moved far from the Σ5(310) GB and Σ29(520) GB, see Figure 7c,d. The results indicate that the GB structure can serve as an effective absorption sink for interstitial atoms and can further affect the distribution of the irradiation-induced defects within the crystal.

**Figure 8:**
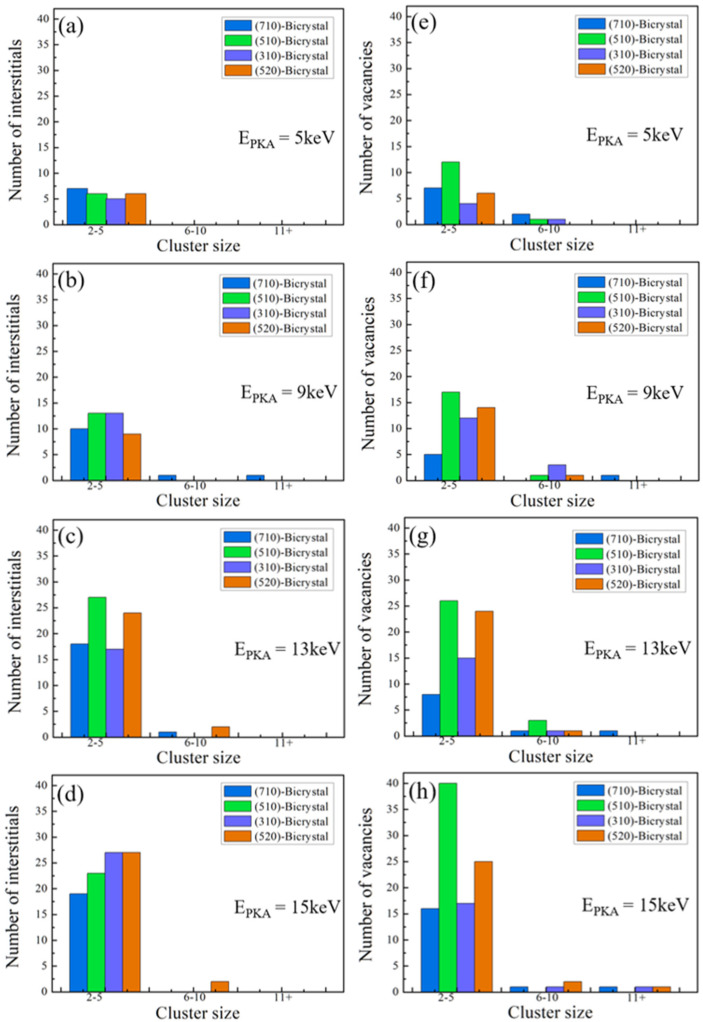
Statistics of interstitial clusters (**a**–**d**) and vacancy clusters (**e**–**h**) with different sizes in bicrystal models at different PKA energies.

During the collision cascade simulations, there is a bias-absorption of interstitial atoms by GBs compared to vacancies. To further investigate the effect of GBs on the distribution of defects, the number of interstitial atoms and vacancies in clusters with different sizes near the GBs at various PKA energies are shown in Figure 8. To present the size of defects clearly, the clusters consisting of 2–5 defects are defined as small size clusters, clusters with 6–10 defects as medium size, and clusters with more than 10 defects as large clusters. Most interstitial atoms form small clusters with the number of interstitial atoms between 2 and 5, while a few large clusters with atoms more than 10 appear as the PKA energy increases. For example, in the case of E_PKA_ = 5 keV, there are no large clusters formed in the crystals (see Figure 8a), but when the PKA energy increases to 9 keV, large clusters are formed near Σ25(710) GB, while there are still no interstitial atoms accumulating into large-size clusters for the other GBs (see Figure 8b). As the energy of PKA further increases from 13 keV to 15 keV, only a few interstitial atoms form medium clusters with atoms between 6 and 10 in Σ13(510) and Σ29(520) GBs, while the other interstitial atoms still form small clusters, as shown in Figure 8c,d. The above simulations show that most of the interstitial atoms were trapped by the GBs and existed as single atom or small clusters. In addition, with the increase of PKA energy, more defects were generated in the crystal, making the probability of large size clusters higher. Similarly, Lin et al. also found that the number of interstitials and vacancies in large-size clusters increased with E_PKA_ in Ni and Ni-Co-Cr-Fe [50].

The number of vacancy clusters generally increases with the increase of PKA energy, as shown in Figure 8e–h. However, the number of small and medium vacancy clusters is more than the interstitial clusters at all PKA energies, and the large-size vacancy clusters are more likely to form at higher PKA energy. In Figure 8f, when the PKA energy increases to 9 keV, the large vacancy clusters are only formed in the case of Σ25(710) GB, while a few vacancies remain, existing as medium clusters in the other three GBs. In Figure 8g, the vacancies form mostly small clusters with a few medium clusters, while the large clusters are found only in Σ25(710) GB at E_PKA_ = 13 keV. As the PKA energy increases to 15 keV, large vacancy clusters are formed in Σ25(710), Σ5(310), and Σ29(520) GB. These results indicate that the Σ25(710) GB has the weakest effect on defect absorption among the four GB structures, which leads to the more likely occurrence of large-size interstitial atoms and vacancy clusters. In comparison, the interstitial atoms only form small-size clusters, but there are some vacancies that form medium clusters when E_PKA_ = 5 keV. No interstitial atoms accumulate into large clusters, while the vacancies form both medium and large-size clusters when E_PKA_ = 15 keV. In other words, compared with the interstitial atoms, the vacancies are more likely to form large-size clusters. The distribution of interstitial clusters tends to be the smaller size due to the bias absorption of interstitial atoms by GBs, while vacancies cannot recombine with the interstitial atoms and remain in the bulk region, which causes more residual vacancies and larger vacancy clusters.

## 4. Conclusions

MD simulations were performed to investigate the effects of temperature, PKA energy, and GB types on the evolution and distribution of irradiation-induced structural defects in tungsten. Four GBs with different structural characteristics were studied; the collision cascades were simulated at the temperature range of 300 to 1500 K, and the PKA energy varied from 1 to 15 keV. The simulation results show that the evolution of structural defects in both single-crystal and bicrystal models under irradiation includes three stages, namely, a rapid increase stage, a thermal spike stage, and a stable stage. The number of defects does not show a regular increase with the increase of temperature; the maximum and stable values of defects at different temperatures fluctuate within a certain range. In contrast, the PKA energy has a significant effect on the number of defects. The defects are positively correlated with energy and with the increase of the PKA energy, more defects are generated and retained. The existence of GBs delayed the recombination of interstitial atoms and vacancies, which caused more defects in the bicrystal than in the single-crystal models at the early stage of the collision cascade. The GBs serve as a defect sink for absorbing interstitial atoms and vacancies, so most of the remaining defects in the bicrystal models are in the form of single atoms or small clusters, and only a few larger clusters are generated at higher PKA energy. The cluster size of interstitials is smaller than vacancies due to the bias segregation of interstitial atoms at GBs.

As the simulation box constructed in this work was small, the energy of PKA could not be selected too high. Therefore, our work mainly focused on the evolution and distribution of point defects. To investigate the process of dislocation generated by high-energy particle irradiation, it is necessary to build a larger model and increase the simulation time. In addition, the GBs constructed in this work were ideal symmetric tilt GBs; the GBs involved in the experiment for research based on the actual situation will be investigated in future work. By exploring the interaction between GBs and defects, we can investigate the relationship between GBs and macroscopic properties of materials, which can help us better guide material processing and production.

## Figures and Tables

**Table 1:** Information of the single-crystal models and bicrystal models. The misorientation angle (θ), the dimensions of the simulation box (X × Y × Z) and the number of atoms (N) are listed.

Models	θ (°)	X × Y × Z (nm)	N
(710)-Bicrystal	16.3	15.560 × 20.001 × 6.28	124,880
(710)-Single	/		126,000
(510)-Bicrystal	22.6	16.073 × 19.878 × 6.28	128,400
(510)-Single	/		128,800
(310)-Bicrystal	36.9	15.967 × 19.938 × 6.28	127,360
(310)-Single	/		128,000
(520)-Bicrystal	43.6	15.306 × 20.408 × 6.28	125,280
(520)-Single	/		125,280

## Data Availability

Please contact the corresponding author for data related to this article.
